# Frequency of Celiac Disease and Spontaneous Normalization Rate of Celiac Serology in Children and Adolescent Patients with Type 1 Diabetes

**DOI:** 10.4274/jcrpe.galenos.2020.2020.0108

**Published:** 2021-02-26

**Authors:** Edip Unal, Meliha Demiral, Birsen Baysal, Mehmet Ağın, Elif Gökçe Devecioğlu, Hüseyin Demirbilek, Mehmet Nuri Özbek

**Affiliations:** 1 Gazi Yaşargil Training and Research Hospital, Clinic of Pediatric Endocrinology, Diyarbakır, Turkey; 2 Gazi Yaşargil Training and Research Hospital, Clinic of Paediatrics, Diyarbakır, Turkey; 3 Gazi Yaşargil Training and Research Hospital, Clinic of Pediatric Gastroenterology, Diyarbakır, Turkey; 4 Gazi Yaşargil Training and Research Hospital, Clinic of Pathology, Diyarbakır, Turkey; 5 Hacettepe University Faculty of Medicine, Department of Pediatric Endocrinology, Ankara, Turkey

**Keywords:** Celiac disease, children, spontaneous normalization, type 1 diabetes

## Abstract

**Objective:**

The prevalence of celiac disease (CD) varies between 1% and 10% in patients with type 1 diabetes mellitus (T1DM). This study aimed to determine the frequency of spontaneous recovery of celiac serology and the biopsy-proven CD (BPCD) frequency in patients with T1DM.

**Methods:**

The data of 668 patients with available celiac serology tests from a total of 779 patients who were followed for the last 10 years with the diagnosis of T1DM were retrospectively evaluated.

**Results:**

Positive serology was detected in 103 out of 668 (15.4%) patients. There was spontaneous normalization in 24 (23.3%), fluctuation in 11 (10.7%) and permanently positive serology in 68 (66%). In 46 out of 53 (86.8%) patients with positive serology and biopsy, CD diagnosis was confirmed by biopsy (BPCD). The frequency of BPCD was 6.9%, and the serology in 76.1% was positive at the time of diagnosis of T1DM. The weight, height and body mass index-standard deviation score at diagnosis were lower in patients with BPCD compared to the group without CD. An anti-tissue transglutaminase-IgA (anti-TTG-IgA) level of 11.8 times the upper limit of normal was the most sensitive (93%) and specific (90%) cut-off for BPCD (area under the curve: 0.95; 95% confidence interval: 0.912-1; p<0.001).

**Conclusion:**

In our cohort, the frequency of positive serology for CD was 15.4%, while the rate of BPCD was 6.9%. The majority (97.8%) of cases were diagnosed within the first five years of T1DM. In 23.3% of cases, positive anti-TTG-IgA spontaneously resolved without a gluten-free diet (GFD). Therefore, serological follow-up instead of immediate duodenal biopsy or GFD therapy, particularly for patients with asymptomatic and mild anti-TTG IgA level, is warranted.

**What is already known on this topic?**

Celiac disease (CD) prevalence varies between 1% and 10% in children and adolescents with type 1 diabetes mellitus (T1DM). In previous reports in about half of the cases, CD was detected at the time of the diagnosis of T1DM. Recently, a few studies have shown normalization of celiac serology in patients with T1DM, even with no gluten-free dietary intervention.

**What this study adds?**

In our study, the majority (97.8%) of cases were diagnosed within the first five years of T1DM. In 23.3% of cases, positive celiac serology spontaneously resolved without a gluten-free diet (GFD). Therefore, considering all of the serologically positive individuals as CD and giving a GFD imposes an additional psychological burden for children and families. This implication would negatively affect the compliance to the T1DM management. The presence of symptoms and high anti-tissue transglutaminase IgA levels were shown to be highly predictive for biopsy-proven CD (BPCD).

## Introduction

Type 1 diabetes mellitus (T1DM), one of the most common chronic diseases in children, characterized by insulin deficiency due to autoimmune destruction of pancreatic beta cells. There is an increased risk of other autoimmune disorders in children with T1DM. The most common autoimmune diseases accompany to T1DM are autoimmune thyroiditis (AITD) and celiac disease (CD) (1). The prevalence of CD in the general population is estimated to be between 0.3% and 1% (2). However, due to increased genetic predisposition, CD prevalence varies between 1% and 10% in children and adolescents with T1DM 3,4,5,6). Since the majority of CD patients can be asymptomatic, screening for CD at the time of T1DM diagnosis is recommended by both American Diabetes Association and the International Society for Pediatric and Adolescent Diabetes (1,7). In seronegative cases at the first screening, if there are no CD symptoms, regular screening every 2-5 years is recommended. However, in patients with CD symptoms or history of CD in first-degree relatives more frequent screening is recommended (1,7). Testing of asymptomatic CD would provide a prompt diagnosis of CD and enable better metabolic control for T1DM patients (8). However, recently, some studies have shown normalization of celiac serology in patients with T1DM, even with no gluten-free dietary intervention. In the mentioned studies, spontaneous normalization developed in 20-35% of the cases (9,10,11). Therefore, considering all of the serologically positive individuals as CD and giving a gluten-free diet (GFD) imposes an additional psychological burden for children and families. This implication would also negatively affect the compliance to the T1DM management.

In the latest European Society for Pediatric Gastroenterology, Hepatology and Nutrition (ESPGHAN) guidelines, it was highlighted that the level of anti-tissue transglutaminase-IgA (anti-TTG IgA) should be at least 10 times higher than the upper limit of normal (ULN) for diagnosis of CD without duodenal biopsy. Rarely, although children with high TGA-IgA (10xULN) levels, they can have normal histopathology. For this reason, it is recommended by EPSGHAN that the diagnosis of CD without biopsy must be confirmed with a positive anti-endomysial antibody (EMA)-IgA test in a second blood sample (12).

The aim of present study was to determine the frequency of biopsy-proven CD (BPCD) and spontaneous resolution of high anti-TTG IgA levels in patients with T1DM. We also investigated the predictive factors for BPCD and spontaneous normalization of celiac serology.

## Methods

The hospital files of 779 patients who have been followed for the last 10 years (2009-2019) with the diagnosis of T1DM at the Pediatric Endocrinology Clinic of Gazi Yaşargil Training and Research Hospital, University of Health Sciences Turkey were retrospectively analyzed. The age, gender, mean glycated hemoglobin (HbA1c) level, and anti-TTG IgA level status of patients with T1DM were recorded. Patients in whom anti-TTG IgA levels were not available were excluded. Anti-TTG IgA level was measured by enzyme-linked immunosorbent assay (Euroimmun kit, Euroimmun Analyzer I, Euroimmun Medizinische Labordiagnostika AG,-23560 Lübeck Germany). Samples were analyzed in a central laboratory where the same method was used for analysis of celiac serology. According to the method used in our laboratory; anti-TTG IgA level <12 IU/mL was considered as negative, 12-18 IU/mL as borderline, and >18 IU/mL as positive celiac serology. Initially positive anti-TTG IgA antibodies that persistently remained negative (<12 IU/mL) for six months was considered as spontaneous normalization of celiac serology (group 1). If anti-TTG IgA level was initially positive, temporarily resolved and then became positive again, this pattern was defined as fluctuation of celiac serology. Pathology reports of all cases who underwent endoscopic biopsy were examined. According to the biopsy results of the patients, those with Marsh scores 2 and 3 were accepted as BPCD (group 2). Those with anti-TTG IgA positive but Marsh score 0 and 1 were considered as potential CD (12).

Serological autoantibody titers were recorded as multiples of the ULN. The threshold value for the ULN was taken as 18 IU/mL. Three times lower than the ULN was considered mild, as ten times higher than the ULN was considered high for anti-TTG IgA level. Bodyweight standard deviation score (SDS), height SDS and body mass index (BMI) SDS values were extracted from the patient medical files. In addition, anthropometric measurements of patients with BPCD were assessed before and during a GFD.

The study was performed in accordance with the Declaration of Helsinki and approved by the Institutional Ethics Committee of Gazi Yaşargil Training and Research Hospital (document number: 17.01.2020/411). Since the study was retrospective, informed consent was deemed unnecessary and not obtained from the parents of the patients.

### Statistical Analysis

Statistical analyses were performed using SPSS for Windows, version 21 (IBM Corp., Armonk, NY, USA). For evaluation of the normality distribution of the data, the Shapiro-Wilk test was used. Numerical variables were expressed as mean±SD or median and interquartile range, categorical variables were expressed as number and per cent (%). For numerical comparisons, independent sample t-test or Mann-Whitney U tests were used subject to the normality distribution of data. Chi-square test was used to compare categorical variables. The repeated measure of weight-SDS, height-SDS and BMI-SDS values of the patients with BPCD at the time of the diagnosis and the last follow-up visit were compared with a paired-sample t-test. In the diagnosis of BPCD, a receiver operating characteristics (ROC) curve analysis was performed for anti-TTG IgA level recorded as multiples of the ULN. A p<0.05 value was considered statistically significant.

## Results

**Figure 1:**
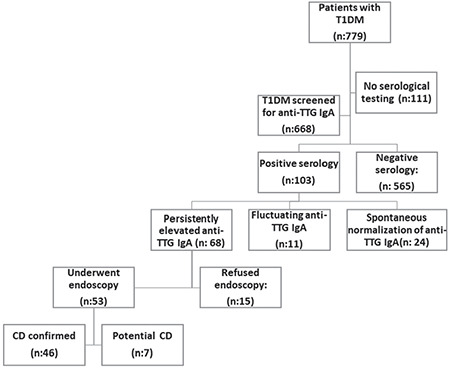
A flow diagram of the study participants T1DM: type 1 diabetes mellitus, CD: celiac disease, anti-TTG IgA: antitissue transglutaminase-IgA

The study included 779 patients, 367 (47.1%) male and 412 (52.9%) female with T1DM. Of those 668 (85.75%) patients had at least one anti-TTG IgA test result (Figure 1). The mean age of diagnosis of T1DM was 8.75±6.75 (range: 0.5-17.92) with a mean follow-up duration of 3.91±4.17 (range: 0.17-16.92) years.

To exclude the false-negative anti-TTG IgA serology due to concomitant IgA deficiency, total serum IgA was measured in all patients undergoing CD serological testing and was within normal limits in all cases. Positive anti-TTG IgA was detected in 103 out of 668 (15.4%) patients. Spontaneous normalization was detected in 24 out of 103 (23.3%) patients within a median duration of nine (range: 3-24 months) months. In the spontaneous normalization group, median follow-up time after the disappearance of anti-TTG IgA antibody was 25.5 months (range: 6-105). There was a statistically significant difference between serum anti-TTG IgA levels of groups 1 and 2 at diagnosis (group 1, median 2 x ULN (range 1.1-11.5) and group 2, median 16.6 x ULN (range 4.1-123) (p<0.05) (Table 1). In one of 24 patients who had spontaneous normalization, the anti-TTG IgA level was above 11 times the ULN. In two of 46 patients who had persistent antibody positivity, the anti-TTG IgA level was below 11 times the ULN.

**Figure 2:**
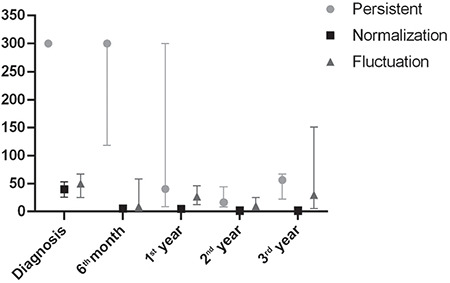
Trend of the anti-tissue transglutaminase-IgA levels in patients with persistent, fluctuation and spontaneous normalization group

In 103 patients with positive celiac serology, fluctuating celiac serology was detected in 11 (10.7%) while celiac serology remained positive in 68 (66%). Autoantibodies became positive again after a median duration of five months (range 3-6 months) in the fluctuation group. The antibody levels of the groups, showing anti-TTG IgA levels of persistent, fluctuation and spontaneous normalization are summarized in Figure 2. An endoscopic biopsy was performed in 53 out of 68 (77.9%) patients who had permanently positive serology. The biopsy was not performed in 15 (22.1%) cases due to family refusal. Forty-six out of 53 (86.8%) patients who underwent biopsy were diagnosed with BPCD suggesting a frequency of BPCD of 6.9% (46/668).

**Figure 3:**
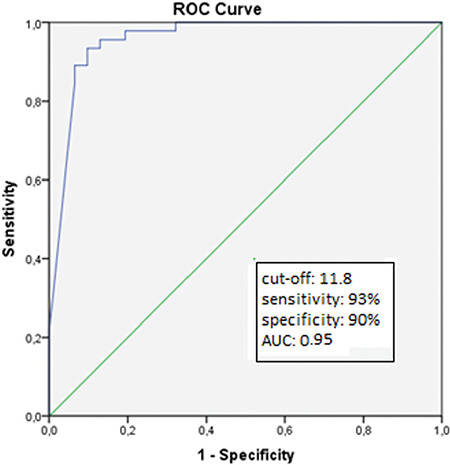
Receiver operating characteristics analysis of antitissue transglutaminase-IgA level for prediction of biopsyproven celiac disease (sensitivity: 93%, specificity: 90%, area under the curve: 0.95, p<0.001) AUC: area under the curve, ROC: receiver operating characteristics

Thirty-five out of 46 (76.1%) patients with BPCD were diagnosed at the time of the diagnosis of T1DM, 11 (21.7%) within following five years and one patient (2.2%) 8.5 years after T1DM diagnosis. Anthropometric measurements were repeated for patients with BPCD before on a GFD and while taking GFD as median of 2.66 years (range 0.25-14.3 years). In BPCD patients, weight (p<0.001), height (p=0.02) and BMI-SDS at the time of the diagnosis (p=0.01) and height-SDS at the final follow-up visit (p 0.001) were found to be significantly lower than the patients who did not have BPCD. There was no statistically significant difference between the mean HbA1c levels of those with BPCD and celiac negative patients (Table 2). In the ROC analysis, an anti-TTG IgA level that was 11.8 times higher than the ULN had the best sensitivity (93%) and specificity (90%) for BPCD (area under the curve: 0.95, 95% Cl: 0.912-1, p<0.001; Figure 3).

Anti-thyroid peroxidase and anti-thyroglobulin serology was examined in 562 of the patients with celiac serology (84.1%) and at least one antibody was positive in 69 cases. While BPCD was present in 8/69 (11.6%) patients with T1DM and positive thyroid autoantibody, it was present in 30/493 (6.1%) patients with negative thyroid autoantibody (p=0.054; Table 3).

BPCD was found in 13/146 (8.9%) patients diagnosed with T1DM under the age of five, and in 33/489 (6.7%) patients over the age of five (p=0.38; Table 3).

The rate of BPCD did not differ between girls at 6.4% and boys at 8.2% (p=0.39; Table 3). There was no statistically significant difference between the weight, height and BMI-SDS values at the time of the diagnosis and the final follow-up visit of patients with BPCD (Table 4).

## Discussion

In the present study, serological CD prevalence was 15.4%, and BPCD prevalence was 6.9%. In patients with T1DM, due to genetic predisposition, the frequency of CD and other autoimmune diseases is higher than the normal population (2). In an international comparative study of 52,721 children and adolescents with T1DM, the overall CD prevalence was reported as 3.5% with a frequency of 1.9% in the USA and 7.7% in Australia (13). This is similar to our study and previous studies conducted in Turkey, which have reported a CD prevalence in children with T1DM of between 3.5% and 7.8% (14,15,16,17).

Recently, some studies evaluating CD prevalence in patients with T1DM, have reported spontaneously normalizing celiac serology in up to 20-35% (9,10,11,18). The duration for a positive serology to become negative was about 1-2 years after diagnosis (10,11). Similarly, in our study in 23.3% of patients, positive celiac serology spontaneously recovered within a median duration of nine months (3-24 months), without GFD intervention. In a study involving 446 pediatric T1DM patients, the rate of spontaneous recovery of celiac serology was reported as 27.6%. Having a negative anti-EMA, and low anti-TTG IgA levels (2.3±2.1 ULN) have been reported as predictive factors (10). In our study, all patients with spontaneously recovered celiac serology were asymptomatic, and median anti-TTG IgA levels were low in the majority. In only one case, the anti-TTG IgA level was 11.5 x ULN. In previous studies, spontaneous recovery of celiac serology in very high anti-TTG IgA levels has not been reported (9,10,11). Therefore, we suggest that serological follow-up might be a more appropriate strategy in patients with asymptomatic and mildly elevated anti-TTG IgA levels instead of performing an intestinal biopsy immediately (9,10,11,18).

In previous studies evaluating spontaneous normalization of celiac serology, there is limited data on re-positivity of celiac serology in patients with spontaneous normalization (9,10,11). In only one study, it was reported that autoantibodies re-appeared in three of 18 patients with spontaneous normalization (10). However, there was no data about the duration for re-appearance (10). In our study, the median follow-up time after anti-TTG IgA level was negative in the spontaneous normalization group was 25.5 months. In three of the 24 patients who with spontaneous normalization of CD serology, the follow-up period while remaining negative was less than one year, while in 21 patients the follow-up period was at least 15 months. Even though the duration of remaining negative was not short, this does not eliminate the possibility of reappearance of CD autoantibodies. Therefore, regular follow-up of celiac serology in patients with spontaneous normalization is warranted.

In the latest ESPGHAN guidelines, it was highlighted that the level of anti-TTG IgA should be at least 10 times higher than the ULN for diagnosis of CD without duodenal biopsy (12). Also, for a serology-based diagnosis without biopsy, human leukocyte antigen testing and the presence of symptoms are not mandatory criteria (12). In our study, the cut-off value of anti-TTG IgA was 11.8 x ULN and shown to have high sensitivity and specificity in predicting BPCD.

Most patients with T1DM and CD have little or no symptoms of malabsorption, and gastrointestinal complaints are usually mild. Therefore, it is challenging to consider a diagnosis of CD in patients with T1DM based on clinical findings or routine laboratory tests. Serological examinations would help to detect subclinical disease (19). In our study, 54.3% of patients with BPCD had gastrointestinal symptoms (abdominal pain, diarrhoea, constipation, distention) or non-gastrointestinal system symptoms (short stature, weight loss, recurrent episodes of hypoglycemia). In a previous study, the presence of CD symptoms, younger age for onset of T1DM, anti-TTG IgA level higher than 7-8 x ULN, and positive anti-EMA were suggested to be predictive for BPCD (11). Similarly, the presence of gastrointestinal symptoms and high anti-TTG IgA levels was shown to be a reliable predictor for CD. In the same study, the endoscopic biopsy was performed in two cases with gastrointestinal symptoms and intermediate levels of anti-TTG IgA (9-16 U/mL), while the biopsy was compatible with CD (20). In our study, the presence of symptoms and high anti-TTG IgA levels were shown to be highly predictive for BPCD. Only in one asymptomatic patient with high anti-TTG IgA level (>10 x ULN), a biopsy was negative, which further emphasized the importance of the presence of CD symptoms.

The overall prevalence of CD is higher in females (21). Various studies in children and adolescents with T1DM reported variable sex distribution; a higher prevalence in girls (10,13,22,23), in boys (3,24) or no difference in boys and girls (9,18,25). In our study, there was no sex predominance of CD prevalence.

The frequency of CD is reported to be higher in patients with an earlier age of T1DM diagnosis (especially <5 years) (5,13,18,21). In contrast, other studies revealed no relationship between the age for diagnosis of T1DM and the frequency of CD (3,19,24,26,27). In our study, there was no statistically significant difference in the frequency of CD between the patients with age for diagnosis of T1DM <5 years and >5 years. In previous reports about half of the cases, the CD was detected at the time of the diagnosis of T1DM diagnosis (9,19), and most of the remaining cases were identified within the first five years following diagnosis of T1DM (9,18). In a review of nine longitudinal cohort studies of celiac screening in patients with T1DM between 5 and 18 years old, it was reported that 79% of celiac cases were diagnosed within the first five years following the diagnosis of T1DM. Therefore, screening for CD is recommended at diagnosis of T1DM and in the subsequent two and five years in case of asymptomatic and negative family history of CD. In the same review, it was mentioned that determination of the frequency of CD after five years of diabetes period is controversial due to a lack of data obtained from long-term follow-up. In some studies with long-term follow-up, 16% of CD cases were reported to be diagnosed between five and 10 years, and 5% after >10 years (28). Thus, CD should be considered at any time in T1DM patients with symptoms of CD (28). In our study, CD was detected in 76.1% of cases at the time of the diagnosis of T1DM, in 21.7% within five years and in 2.2% of the cases 8.5 years following the diagnosis of the T1DM. To the best of our knowledge, the rate of detection CD at the time of the diagnosis of T1DM (76.1%) is the highest ever reported in the literature.

The comorbidity of CD and T1DM in children has been reported to be associated with an increased risk of AITD (29,30,31). However, although studies evaluating CD prevalence in patients with both T1DM and AITD are scarce, the few studies conducted have revealed no difference (32,33). In our study, CD prevalence in patients with T1DM and AITD (11.6%) was higher than in patients with T1DM alone (6.1%), but the difference did not reach statistical significance.

There are controversial data regarding metabolic control and its association with T1DM and CD comorbidity. Some studies have reported no difference in metabolic control between children with T1DM only and children with concomitant T1DM and CD (34,35,36), while in some studies, HbA1c was lower in patients with T1DM and CD comorbidity (37). In the present study, we did not find a difference in HbA1c levels of T1DM patients with and without CD. However, it should be kept in mind that having a relatively acceptable HbA1c concentration does not eliminate the risk of developing diabetes complications. In addition, CD may increase glycemic variability and frequent hypoglycemia due to malabsorption which may result in a low HbA1c, thereby undersetimating the degree of glycemic control.

It has been shown that there was no difference in height and BMI SDS scores between children with a diagnosis of T1DM only and children with both T1DM and CD (19,37,38). However, some studies reported a lower height SDS in T1DM patients with CD (13,39). There are also studies indicating that GFD therapy does not affect height and BMI SDS (37,38,40), while some others reported a better height SDS after GFD (41). In our study, the height, weight and BMI SDS values of T1DM patients with CD were lower than those without CD. In addition, we did not find any difference between the weight, BMI and height SDS of the patients with CD before and after the GFD. This finding was in line with some previous reports (37,38). However, the lack of improvement in growth parameters may be attributed to non-compliance with GFD due to the low socioeconomic and cultural level of the region where our study was conducted.

### Study Limitations

The main limitation of our study was that some individuals with positive TTG-IgA antibodies (n=15) did not undergo duodenal biopsy. Another major limitation of the study is the retrospective nature of design. It was also a limitation that anti-EMA were not checked.

## Conclusion

The frequency of BPCD in our patients with T1DM was 6.9%. Approximately three quarters of the cases were diagnosed at the time of diagnosis of T1DM and 97.8% were diagnosed within the first five years. High anti-TTG IgA titers, particularly in patients with CD symptoms, can be used as a valuable parameter to predict CD. However, spontaneous normalization of celiac serology suggested performing serological follow-up instead of immediate duodenal biopsy or GFD therapy, especially in patients with asymptomatic and mild anti-TTG IgA antibody levels. Having CD at the time of diagnosis of T1DM did not affect the metabolic control whilst being associated with poor growth parameters. Nevertheless, no improvement was seen in growth parameters which were attributed to non-compliance to GFD.

## Figures and Tables

**Table 1:**
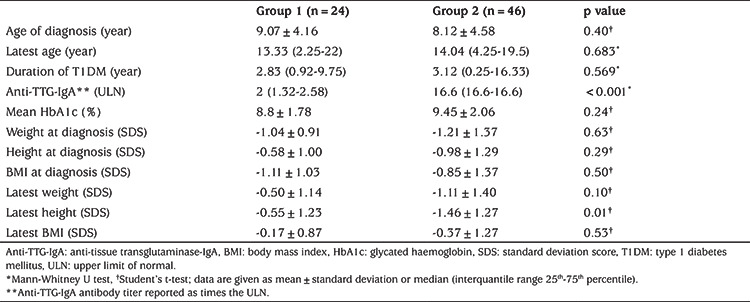
Comparison of anthropometric and laboratory features of type 1 diabetes mellitus patients with biopsy-proven celiac disease (group 2) and spontaneously recovered positive celiac serology (group 1)

**Table 2:**
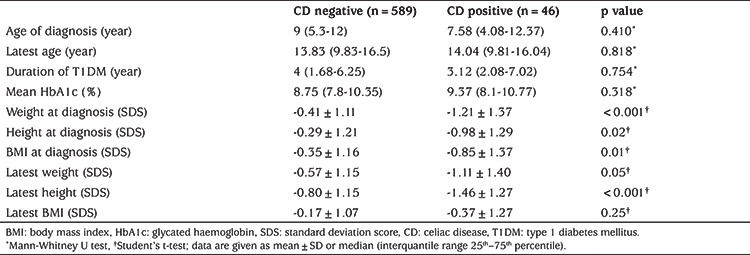
Comparison of anthropometric and laboratory features of type 1 diabetes mellitus patients with and without biopsy-proven celiac disease

**Table 3:**
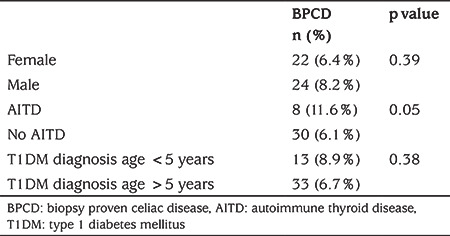
The frequency of biopsy-proven celiac diseaseaccording to age and presence of autoimmune thyroid disease accompanying type 1 diabetes mellitus

**Table 4:**
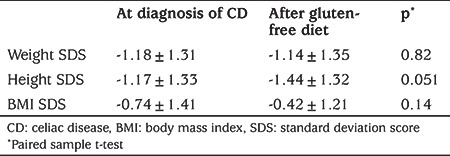
Comparison of anthropometric features of type 1 diabetes mellitus patients at the time of the diagnosis of celiac disease and during gluten-free diet at follow up
